# Influence of Nitrogen Sources Applied by Fertigation to an Enriched Soil with Organic Compost on Growth, Mineral Nutrition, and Phytochemicals Content of Coriander (*Coriandrum sativum* L.) in Two Successive Harvests

**DOI:** 10.3390/plants11010022

**Published:** 2021-12-22

**Authors:** Rui M. A. Machado, Isabel Alves-Pereira, Yasmin Faty, Sara Perdigão, Rui Ferreira

**Affiliations:** 1 MED—Mediterranean Institute for Agriculture, Environment and Development, Departamento de Fitotecnia, Escola de Ciências e Tecnologia, Universidade de Évora, 7002-554 Evora, Portugal; 2 MED—Mediterranean Institute for Agriculture, Environment and Development, Departamento de Química e Bioquímica, Escola de Ciências e Tecnologia, Universidade de Évora, 7002-554 Evora, Portugal; raf@uevora.pt; 3 Departamento de Química e Bioquímica, Escola de Ciências e Tecnologia, Universidade de Évora, 7002-554 Evora, Portugal; yasminfaty@hotmail.com (Y.F.); saragalhofoperdigao@gmail.com (S.P.)

**Keywords:** sustainable agriculture, shoot nutrient content, total phenols, proline, FRAP, DPPH

## Abstract

The aim of the present study was to evaluate the effects of nitrogen source applied by fertigation to an enriched soil with organic compost on plant growth, mineral nutrition, and phytochemical contents in two successive harvests in coriander. The treatments were as follows: unfertilized soil, soil enriched with organic compost, and soil enriched with organic compost to which 60 kg N ha^−1^ as ammonium nitrate and as ammonium sulfate applied by fertigation were added. Ammonium nitrate addition allowed to obtain a high total fresh yield (3.6 kg m^−2^) with a low inorganic nitrogen input. Ammonium nitrate increased plant shoot dry weight; fresh yield; and shoot N, K, and Ca uptake in the first harvest. Ammonium nitrate relative to organic compost and to ammonium sulfate increased fresh yield by approximately 57 and 25%, respectively. However, ammonium sulfate in the first harvest greatly increased shoot total phenols, from 137 mgGAE/100 g FW in ammonium nitrate to 280.4 mgGAE/100 g FW. Coriander’s fresh yield, in the second harvest, was unaffected by nitrogen addition. However, ammonium nitrate increased shoot total phenols and FRAP activity. Overall, the shoot phytochemical accumulation in the second harvest was lower than in the first. The combined application of ammonium nitrate and organic compost is a strategy to reduce inorganic nitrogen application.

## 1. Introduction

Coriander, *Coriandrum sativum* L., is an herbaceous annual vegetable, belonging to the Apiaceae family. It is native from the Mediterranean and middle east regions, nowadays being widely cultivated worldwide, for its leaves and or fruits [1]. The leaves are used as fresh, frozen, or dehydrated condiments [2]. In the Alentejo region of Portugal, coriander leaves are the most widely used aromatic ingredient in soups, sauces, salads, açordas (bread soup), and so on [3]. Coriander leaves contain high-level minerals and phytochemicals [4,5].

Nitrogen fertilization of coriander as the other crops is essential to plant growth [6,7]. Moreover, it may also affect phytochemical accumulation owing to rate, form, nitrate/ammonium ratio, method of application, and effects on soil pH. Nitrogen deficiency may pose stresses on plants that can devote resources toward antioxidants’ syntheses. Nitrogen deficiency may increase total phenols’ concentration in plants [8,9,10,11]. Lower levels of phenolic, flavonoid, and anthocyanins compounds in plants grown under high N supply have been reported by [12,13,14]. In spinach [15] and mustard (*Brassica juncea*) [16], nitrogen addition decreased total phenols’ content.

Currently, one of the major challenges in vegetable production is to reduce inorganic nitrogen fertilization and obtain a high yield, which can be achieved by increasing nitrogen use efficiency. Efficient use of nitrogen from the different sources (organic or inorganic nitrogen) is the basis for mitigating negative environmental impacts of the emission of nitrous oxide (N_2_O) and carbon dioxide (CO_2_) to air and nitrate (NO_3_^−)^ leaching to surface and ground waters. Organic compost as a unique source of nutrients did not satisfy plant nutrient requirements, mainly nitrogen, which can drastically reduce crop yield and affect the quality [15,17]. One of the strategies can be the combined application of organic compost with inorganic nitrogen at levels lower than usual [18], applied by fertigation [15]. In spinach, this allowed to reduce the inorganic nitrogen addition, obtain high yields, and increase soil fertility [17]. Moreover, it may increase nitrogen use efficiency and contribute to the circular economy and mitigation of environmental impacts associated with inorganic nitrogen fertilizers.

Coriander can be planted by direct sowing or by transplanting the seedlings. Despite the cost of the seedling plantings, the transplanting method is increasing, as it is a reliable method to improve growth, achieve earliness, higher yield, plant uniformity at harvest, and so on. To reduce the cost of the seedlings, an alternative to a single harvest may be to do two harvests.

This study aimed to analyze the effect of inorganic nitrogen source applied by fertigation to an enriched soil with organic compost on plant growth, mineral nutrition, and the quality of coriander transplanted in two consecutive harvests.

## 2. Results and Discussion

### 2.1. Shoot Dry Weight and Fresh Yield

The addition of organic compost increased the fresh yield compared with the control (unfertilized soil) (Table 1). In this treatment, the plants after the first ten days did not grow and began to wither and die. This was due to nutrient deficiency, increased by the high plant density (316 plants/m^2^). The interaction between OC plus nitrogen and the harvest on shoot dry weight and fresh yield was significant (*p* < 0.001), which indicates that the plant growth response to nitrogen addition to compost differed among harvests (Table 1). While in the first harvest, there was a positive effect between inorganic nitrogen addition and the fresh yield, in the second harvest, that effect was not observed.

In the first harvest, coriander shoot dry weight and fresh yield increased significantly with inorganic nitrogen addition to organic compost (Table 1). However, plants grown with ammonium nitrate had greater shoot dry weight (0.72 g/plant) and fresh yield (2.43 kg m^−2^) than those grown in the other ammonium. For the same coriander cultivar, in the first harvest, shoot dry weight average values were higher than those reported by Donega et al. [19] (0.38 mg/plant). Ammonium nitrate addition compared with organic compost or organic compost plus ammonium sulfate increased fresh yield by approximately 57 and 25%, respectively.

In the second harvest, plants grown with ammonium sulfate had a lower shoot dry weight than the other plants grown with ammonium sulfate or with organic compost (Table 1). However, coriander shoot fresh yield was not significantly affected by the addition of nitrogen (Table 1).

Therefore, nitrogen addition increased the yield, but its addition after the first growing period has maybe been unnecessary. This can be because of the higher availability of nitrogen from compost mineralization and/or lesser nitrogen uptake by plants (Table 3). Therefore, in further research, the effect of the rate of nitrogen in the second season must be studied.

### 2.2. Shoot Nutrient Concentration and Uptake

Shoot macronutrient concentration (except P) was not affected by the interaction of the treatments (Table 2), meaning that the effect of the addition of nitrogen to organic compost on nutrient concentration has not differed among harvests. In both harvests, shoot N content increased with nitrogen application, but it was not affected significantly by nitrogen source (Table 2). However, shoot nitrogen uptake increased significantly in both harvests when the nitrogen source was ammonium nitrate (Table 3).

In the second harvest, average shoot nitrogen concentrations in plants grown with nitrogen were higher than in those in the first harvest, ranging from 6.68 to 6.76 mg g^−1^. These concentrations were slightly higher than the higher end of the range considered to be sufficient (4 to 6 mg g^−1^) by [20].

Shoot P in the first harvest increased with nitrogen addition, while in the second harvest, it decreased, probably because of P fixation caused by acidification of the soil. The addition of the same sources of nitrogen to compost on the same soil decreased soil pH [15].

In both harvests, shoot K, Ca, and Mg contents were not significantly affected by the addition of nitrogen (Table 2). However, in the first harvest, nitrogen addition increased shoot K, Ca, and Mg uptake (Table 3).

Shoot N, P, Ca, and Mg contents were higher in the second harvest than in the first. This may be because of the higher availability of these nutrients in soil solution owing to compost mineralization and/or the physiological age of the tissues. Concentrations of elements in leaves vary with physiological age [21]. In the present study, despite the leaves in both harvests having a similar size, the plants can were collected in different stages as the growth periods before the harvests were different (31 and 18 days, before the first and second harvest, respectively).

In first harvest, shoot Zn concentration was higher in plants grown with ammonium sulfate than those grown with compost or compost and ammonium nitrate (Table 2). The addition of ammonium nitrate to compost led to a decrease in shoot Mn and Cu contents.

In the second harvest, shoot Mn and Zn contents increased with ammonium sulfate addition. That is probably related to increasing Mn and Zn availability in soil solution due to ammonium nitrification and/or its uptake by plants, which decreases soil pH. For the same rate of N added, ammonium sulfate has a greater potential to produce hydronium ion (H_3_O^+^) than ammonium nitrate. All the nitrogen in ammonium sulfate may be converted to nitrate, while in ammonium nitrate, only half can be converted [22]. On the other hand, ammonium uptake is known to involve proton extrusion to the rhizosphere. Conversely, ammonium nitrate has a smaller pH reduction effect because, when plants take up nitrate, the roots excrete bicarbonate (HCO_3_^−^) and/or hydroxyl (OH^−^) ions into the soil, which increases pH [23]. Ammonium sulfate addition combined with organic compost decreased the soil pH more than ammonium nitrate [15].

Shoot Mn and Zn in the second harvest in plants grown with inorganic nitrogen was much higher than in the first harvest. This can be because of the accumulated effect of the ammonium application on pH. Shoot Mn (302 μg g^−1^ DW) and Zn (201 μg g^−1^ DW) contents were higher in plants grown with ammonium sulfate than those grown in the other treatments. Despite the higher value of shoot Mn and Zn in the plants grown with ammonium sulfate, none of those plants showed visual symptoms of Mn or Zn toxicity. Average shoot Zn concentrations ranged from 182.5 to 200.5 µg g^−1^ DW. These values were lower or equal to the low end of the range of the values that can inhibit the growth of most plants (200–500 µg g^−1^ DW [24].

Shoot Fe and B were not significantly affected by the addition of nitrogen to compost. Ammonium nitrate addition in the first harvest significantly decreased shoot Cu, while in the second harvest, it led to an increase in Cu content in leaves. This may be related to changes in soil pH and/or nitrogen form concentration in the rhizosphere. In wheat, the authors of [25] reported that the antagonism between Cu and N was greater with ammonium-N (NH_4_^+^) form than with nitrate-N (NO_3_^−^).

In the first harvest, the addition of both inorganic nitrogen sources to organic compost significantly increased shoot N, P, K, and Ca uptake. However, the addition of ammonium nitrate led to a high increase in N, K, and Ca uptake. This indicates that ammonium nitrate nutrition enhanced coriander shoot macronutrient uptake.

Average shoot nitrogen uptake values ranged from 21.7 and 38.9 mg/plant. These values were higher or similar than that reported by [19] to the same cultivar (22.47 mg N/plant). Moreover, the range of values of P (2.57 mg P/plant), K (36.89 mg K/plant), Ca (3.28 mg Ca/plant), and Mg (2.37 mg Mg/plant) uptake were higher than or similar to those reported [19].

In the second harvest, the addition of ammonium nitrate to compost increased shoot N, K, Ca, and Mg relatively to the plants grown with compost plus ammonium sulfate (Table 3). Shoot K, Ca, and Mg uptake by plants grown only with organic compost was not significantly different from those grown with ammonium nitrate (Table 3).

In both harvests, the plants grown with the addition of ammonium sulfate uptake less N, K, and Ca than those plants grown with ammonium nitrate. That can be because of the fact that NH_4_^+^ assimilation may compete with K and Ca [21,26,27]. Therefore, the results indicate that, from the standpoint of mineral nutrients’ accumulation, ammonium nitrate application is advantageous and that, after the first harvest, inorganic nitrogen addition must be reduced.

### 2.3. Photosynthetic Pigments

Leaf photosynthetic pigments, except carotenoids, were not significantly affected by the interaction between fertilizer treatments and harvest. In both harvests, plants grown with inorganic nitrogen had a higher content of leaf Chl a, b, and total chlorophyll (a + b) than those grown only with organic compost (Table 4). However, in the second harvest, total Chl and Chl a were higher in plants grown with ammonium nitrate than those grown with ammonium sulfate (Table 4).

In the second harvest, total leaf chlorophyll and Chl a, in each treatment, were higher than in the first harvest (Table 4), which may be due to an increase in shoot N content (Table 2). Indeed, leaf Chl a increased linearly with the shoot nitrogen content (leaf Chl a = 24.64 shoot N = 53.7; R^2^ = 0.83, *p* ≤ 0.001).

The total leaf chlorophyll average values ranged from 96.41 to 140.52 and from 121.48 to 187.14 mg/100 g FW in the first and second harvest, respectively. These values were higher or were within the range than those reported [28] (from 79 to 116 mg/100 g FW) in coriander plants harvested 18 days after transplanting and higher than those reported by [29] (from 24.5 to 80.8 mg/100 g FW).

Leaf Cc was affected by interaction organic compost plus nitrogen and harvest, meaning that the effects of nitrogen addition to compost on leaf Cc content differed among the harvest. In the first harvest, leaf Cc content increased with inorganic nitrogen addition (Table 4). The increase in Cc with nitrogen addition was also reported in vegetable crops [17,30,31]. In red and green lettuce in general, the concentration of chlorophylls and carotenoids decreased with decreasing nitrogen concentration [32]. In parsley, lutein-zeaxanthin and ß-carotene increased with the increase of nitrogen in the nutrient solution [33]. Leaf Cc was positively correlated with shoot nitrogen uptake (r = 0.79, *p* <0.001).

However, in the second harvest, leaf Cc was not affected by nitrogen addition to organic compost despite leaf nitrogen uptake increased with ammonium nitrate addition (Table 3).

Leaf Cc average values ranged from 41.79 to 83.42 mg/100 g FW. These values were lower than those reported by [34] (169 mg/100 g FW), but higher than those reported by [29] (6.9 mg/100 g FW) and [35] (12.1 mg/100 g FW). The differences can be due to the factors that affected carotenoid accumulation such as abiotic factors (e.g., light and temperature) [36].

### 2.4. Phytochemical Accumulation and Antioxidant Activity

Phytochemical accumulation and antioxidant activity were significantly affected by the interaction between OC plus nitrogen and the harvest. This indicates that the response of phytonutrient content to nitrogen addition to compost differed among harvests (Table 5).

#### 2.4.1. Total Phenols

In the first harvest, shoot TPC was significantly affected by the source of inorganic nitrogen. Shoot TPC was lower in plants grown with ammonium nitrate than those grown with compost or compost and ammonium sulfate (Table 5). The decrease in TPC with nitrogen addition was also reported in different plants [15,16,37,38,39]. However, plants grown with ammonium sulfate had higher TPC than those grown only with compost and compost plus ammonium nitrate. This may be because of the effects of ammonium sulfate in soil pH, nitrogen form (NH_4_^+^), and/or sulfur present in the fertilizer. In different crops, an increase in TPC due to NH_4_^+^ addition was reported [39,40,41,42,43]. In mustard, sulfur increased TPC [16].

On the contrary, in the second harvest, plants grown with ammonium nitrate had higher shoot TPC than those grown with compost and compost plus ammonium sulfate. In this harvest, nitrogen addition in both sources to compost did not decrease shoot TPC. This is not in agreement with the commonly reported decrease in the TPC content with nitrogen addition. However, a major part of those results was obtained in plants grown in hydroponic solutions where nitrogen form and pH remain constant, which does not happen on soil enriched with organic compost. In plants grown in soil [44,45], also report a shoot TPC increase with nitrogen supply. In *Allium fistulosum* L., grown in soil [46], also reported a non-linear increase in total phenols’ content with nitrogen increase.

Regardless of the treatment in the second harvest, shoot TPC contents were lower than in the first harvest.

#### 2.4.2. Ascorbate

Inorganic nitrogen addition only affected shoot ascorbate (AsA) in the first harvest (Table 5). It was higher in plants grown only with compost than those grown with inorganic nitrogen addition to compost. Average values of AsA content in the plants of the second harvest were 1.5 to 2.0 times greater than in the first harvest. That could be because of the stage of growth because, as previously mentioned, plants may have been collected in different stages of growth. Ascorbate content is influenced by the crop development stage [47,48]. In baby spinach, the authors of [48] reported a decrease in shoot AsA with the advance in plant growth.

Average shoot AsA values ranged from 11.5 to 15.1 and from 22.4 to 23.0 mg/100 g FW in the first and second harvest, respectively. These values were lower than those reported by [29] (35.0 mg/100 g FW) and lower or within the range of values reported by [49] (14 to 40 mg/100 g FW) and [50] (20.4 to 40.5 mg/100 g FW).

#### 2.4.3. Proline and Proline Dehydrogenase Enzyme Activity (PDH)

Shoot proline, in the first harvest, was lower in plants grown with ammonium nitrate than those plants grown with compost or compost + ammonium sulfate (Table 5). This indicates that plants grown with ammonium nitrate were subject to lower stress conditions because the accumulation of this amino acid is closely related to abiotic stress, which may contribute to a higher yield and shoot biomass (Table 1). However, in the second harvest, shoot proline was not affected by nitrogen addition.

The different response to nitrogen addition between harvests may be due to plant N uptake, as L-proline is a good nitrogen-storage compound whose metabolism depends on nitrogen uptake [51]. Nitrogen content and its level in plants are correlated with the L-proline metabolism and L-proline level as well [52]. In the second harvest, shoot N uptake was not influenced by the treatments (Table 5).

In mustard, nitrogen differentially regulates proline production and ethylene formation to alleviate the adverse effect of salinity on photosynthesis [53].

In both harvests, shoot proline dehydrogenase (PDH) activity was affected by nitrogen addition. Ammonium sulfate addition in both harvests significantly increased PDH activity (Table 5). In the second harvest, PDH level was lower in plants grown with compost plus ammonium nitrate.

PDH is also sensitive to environmental stresses. Abiotic injuries repress PDH and stimulate Pro synthesis, thus leading to net Pro accumulation. Once the stress is relieved, PDH becomes activated to consume the stored Pro [54,55,56,57].

#### 2.4.4. DPPH-Radical Scavenging Activity and FRAP-Ferric-Reducing Antioxidant Activity

Shoot DPPH and FRAP were significantly affected by the interaction between OC plus nitrogen and the harvest (Table 5). In the first harvest, plants grown with ammonium sulfate had lower shoot DPPH than in those grown with compost, and those grown with compost plus ammonium nitrate (Table 5). This has also been reported in spinach [15].

In the second harvest, shoot DPPH was not affected by nitrogen addition. The DPPH antioxidant activity in the leaves in the second harvest, in the treatments’ organic compost, and in organic compost plus ammonium nitrate was significantly lower than those of the same treatments in the first harvest (Table 5).

In the first harvest, shoot FRAP was not affected by inorganic nitrogen. However, in the second harvest, shoot FRAP increased significantly with ammonium nitrate addition. Regardless, the treatment shoot FRAP was lower in the second harvest.

The shoot FRAP of the two harvests was strongly correlated with total phenols’ content (r = 0.84, *p* < 0.001). In coriander, antioxidant activity was also related to total phenol [58]. The correlation between TPC and antioxidant activity measured by FRAP has also been reported in spinach [59] and mustard [16]. In both harvests, ammonium nitrate addition to compost increased or maintained antioxidant activity measured by DPPH and FRAP assays.

Overall, shoot phytochemical compounds content (except shoot AsA) decreased in the second harvest. This was also reported in parsley [60] and basil [61]. In *Cichorium spinosum*, the decrease in phytochemical accumulation only occurred after the second harvest [40].

The decrease in the phytochemical accumulation may be due to plant physiology (e.g., the stage of growth or degree of maturation) and/or environmental conditions, among others.

Some studies have reported that phytochemical accumulation is affected by the stage of growth. The chemical composition and bioactive compounds content of leaves varies with plant development [40,62]. In the present study, as previously mentioned, plants may have been collected in different crop stages of growth. The environmental conditions were different; the solar radiation decreased after the first harvest, which can lead to a decrease in the synthesis of phenolic compounds and, consequently, antioxidant activity [63,64]. Plant secondary metabolites’ accumulation is strongly dependent on a variety of environmental factors [65].

Therefore, future studies are needed to analyze whether the decrease in phytochemical accumulation in the second harvest is due to internal plant factors and/or abiotic fac-tors.

## 3. Materials and Methods

### 3.1. Growth Conditions

**Figure 1:**
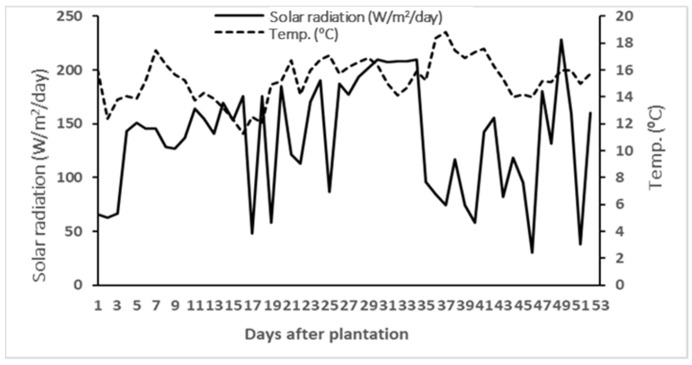
Average air temperature inside the greenhouse at plant level and solar radiation outside the greenhouse during the experimental period. Solar radiation data were obtained from an automated weather station located at Mitra research station [66].

A pot experiment was conducted in a greenhouse located at the “Herdade Experimental da Mitra” (38°31′52″ N; 8°01′05″ W), University of Évora, Portugal. The greenhouse was covered with polycarbonate and had no supplemental lighting. The average air temperatures inside of the greenhouse ranged from 12 to 19 °C (Figure 1), and solar radiation ranged from 34 to 228 W·m^−2^·day^−1^.

The experiment was carried out in plastic pots. Each 12 L plastic pot (21 cm high × 27 cm diameter) was filled with 14 kg of luvisol sandy loam soil obtained from the upper 40 cm soil of the Mitra Research Farm in Évora, Portugal. The soil presented 1.5% organic matter content, a bulk density of 1.3 g·cm^−1^, a pH of 7.2 (1 soil: 2.5 water), an ECe of 0.25 dS m^−1^, 35 mg NO_3_-kg^−1^, 158 mg -K·kg^−1^, 162 mg P·kg^−1^, 7.57 meq Ca^2+^/100 g, and 1.67 meq Mg^2+^/100 g.

Ten days prior to transplanting, 86 g of a commercial organic compost of the manure of the poultry, sheep, and horses, from controlled aerobic fermentation (Biofertil N6, Nutrofertil Nutrição e Fertilizantes, Lda, Portugal) in pellets, certified for organic farming was added to each pot and mixed with the upper 10 cm of the soil.

The physicochemical characteristics of the compost were as follows: organic matter (52%), moisture (12%), total carbon (30.5%), pH (6.5), electrical conductivity (6.4 dSm^−1^), total nitrogen (6.4%), organic nitrogen (6.4%), P_2_O_5_ (2.5%), K_2_O (2.4%), CaO (8.4%), MgO (0.3%), B (0.0020%), and a C/N ratio of 5.

Four treatments were carried out: unfertilized soil (S), organic compost (OC), organic compost + 60 kg of N ha^−1^ applied as ammonium sulfate (21% NH_4_–N and 60% SO_3_) (OC + AS), and organic compost + 60 kg N ha^−1^ applied as ammonium nitrate (16.9% NO_3_–N and 16.7% NH_4_–N) (OC + AN). Treatments were arranged in a randomized complete block design with six replicate pots per treatment.

At each pot of the treatments, OC, OC + AS, and OC + AN were added through the compost 4.84, 1.89, 1.81, 6.36, and 0.23 g pot of N, P_2_O_5_, K_2_O, CaO, and MgO, respectively. Soil blocked coriander (*Coriandrum sativum* L. cv. Santo) seedlings (six seedlings per block), three blocs per pot (316 planta m^−2^), were transplanted (on 23-01-2018) 30 days after emergence into pots.

Inorganic nitrogen was applied by fertigation, weekly, in five equal fertilizer applications, three before the first harvest and two between that and the second harvest.

### 3.2. Measurements

#### 3.2.1. Plant Growth and Mineral Content

Coriander was harvested at 38 (26 February 2018) and 51 (15 March 2018) DAT, respectively. The shoots of the plants in the first harvest were cut off above the apical meristem. The leaves were washed, oven-dried at 80 °C for 3 d, ground so that they would pass through a 40-mesh sieve and analyzed for N, P, K, Ca, Mg, S, Fe, B, Cu, Mn, and Zn. Total N was analyzed by using a combustion analyzer (Leco Corp., St. Josef, MI, USA). The K was analyzed by flame photometry (Jenway, Dunmow, UK). P and B were analyzed using a UV/Vis spectrometer (Perkin Elmer lamba25). The remaining nutrients were analyzed using an atomic absorption spectrometer (Perkin Elmer, Inc., Shelton, CT, USA).

#### 3.2.2. Phytochemical Accumulation and Antioxidant Activity

For determining the photosynthetic pigments’ level, 1.000 *g* of Leaf-blade from each treatment (five replicates) was macerated in a mortar and immediately homogenized in 8 mL of methanol/water solution (90:10 (*v*/*v*), MW90-extract) for 1 min and then centrifuged at 4 °C at 6.440× *g* for 5 min.

Aliquots of MW90-extract were stored at −20 °C for later determination of chlo-rophyll a (Chl a) and b (Chl b) and total carotenoids (Cc) in accordance with the method of Lichtenthaler and Buschmann [67].

To determine the content of total phenol compounds (TPCs), ascorbate (AsA), and proline (Pro), as well as antioxidant activity (FRAP, DPPH), a sample of 1.000 g of leaf-blade from each plant from the four treatments (five replicates) was macerated in a mortar and homogenized in 8 mL of methanol/water solution (80:20 (*v*/*v*) MW80-extracts) for 1 min and then centrifuged for 5 min, at 4 °C at 6.440× *g*. Aliquots of the MW80-extracts were stored at −20 °C for later use.

The content in TPC was determined in accordance with Bouayed et al. [68] using Folin–Ciocalteau’s phenol reagent and reading the absorbance at 760 nm. TPC content ex-pressed as milligram of gallic acid equivalents (GAE) per 100 g of fresh weight (FW) was calculated from a freshly prepared calibration curve (GAE, n = 6 concentrations from 0 to 50 mg/L).

For determination of AsA content, each sample (extracts or standard suitably di-luted) was incubated in a mixture containing 5% trichloroacetic acid in ethanol, 0.4% H_3_PO_4_, 0.5% β-phenanthroline in ethanol, and 0.03% FeCl_3_ in ethanol and warmed at 30 °C for 90 min [69]. The absorbance of Fe(II)–β-phenanthroline complex formed was read at 534 nm. AsA concentration was calculated from a freshly prepared calibration curve (ascorbic acid, n = 6 concentrations from 0 to 30 mg/L).

Free Pro level of MW80-extracts was determined using the acid ninhydrin reaction with the amino acid, reading the absorbance of formed formazan at 546 nm. The concentration of Pro was calculated using a calibration curve prepared from standard solutions of pure proline (L-proline, n = 6 concentrations between 0 and 20 mg/L) [70].

Free radical scavenging antioxidant activity (DPPH) was determined by measuring the ability of coriander leaf-blade extracts to convert the stable organic radical DPPH^•^ (2, 2-diphenyl-1-picryl-hydrazyl) of violet color into a stable product, DPPH-H (diphenyl-picryl hydrazine) of yellow color. Thus, aliquots of an extemporaneous methanolic solution of 0.03 g/L DPPH^•^, kept in the dark, were reacted with a sample or a standard solution. The reduction of DPPH^•^ to DPPH-H was followed by reading the absorbance at 515 nm, at 25 °C, for 180 s. Antioxidant activity reported as milligram of GAE per 100 g of FW was calculated using a calibration curve (GAE, n = 8 concentrations from 0 to 200 mg L^−1^) [71].

For determination of the reducing power of iron (FRAP) present in the coriander extracts, 0.050 mL of the sample (plant extracts) or standard was mixed with 0.950 mL of FRAP reagent and the absorbance change was read at 593 nm, at 37 °C, for 180 s. FRAP reagent was freshly prepared by mixing 300 mM acetate buffer pH 3.6, 10 mM TPTZ solution in 40 mM HCl, and 20 mM iron (III) chloride solution (10:1:1, *v*/*v*/*v*) at 37 °C. The antioxidant activity reported as milligram of Trolox equivalents per 100 of FW was calculated using a calibration curve (Trolox solution, n = 8 concentrations from 0 to 1120 mg L^−1^) [61]. For all determinations, a Genesys10S UV/Vis spectrophotometer was used.

To determine water-soluble protein content and proline dehydrogenase (PDH) enzyme activity samples of 0.2500 g of coriander leaves were macerated in the presence of liquid N_2_ and homogenized in 50 mM phosphate buffer pH 7.0. The supernatant obtained by means of the centrifugation of this extract for 15 min at 15,000× *g* at 4 °C was collected and stored in aliquots at −20 °C (PB-extract) for later use [72,73].

PDH enzyme activity was assayed in accordance with Costilow [72] following the reduction of NAD^+^ at 340 nm at 30 °C during 180 s, in a double-beam Hitachi-U2001 spectrophotometer with temperature control. The reaction was initiated with the addition of 2 mM L-proline to an assay mixture containing 100 mM Na_2_CO_3_-NaHCO_3_ buffer pH 10.3, 10 mM NAD^+^, and the PB-extract. The control cuvette contained all the solutions except NAD^+^. Enzyme activity was calculated based on the reaction curve slope, using the extinction coefficient of ε = 6.22 mM^−1^ cm^−1^. PDH activity was expressed in nmol min^−1^/mg protein. The protein content of the PB-extract was determined in accordance with the Lowry method [74], using a calibration curve (bovine serum albumin, BSA; n = 6 concentrations from 0 to 200 mg/mL).

### 3.3. Data Analysis

Data were processed by means of the variance analysis using SPSS Statistics 24 software (Chicago, IL, USA). Means were separated at the 5% level using Duncan’s new multiple range test. Bivariate correlation analysis between parameters was realized using Pearson’s bilateral correlation coefficient.

## 4. Conclusions

The two consecutive harvests increased the yield, and this was greater with nitrogen addition to compost. However, the addition of ammonium nitrate led to an increase in N, K, and Ca uptake and a fresh yield approximately 25% higher versus ammonium sulfate. The addition of ammonium sulfate in the first harvest substantially increased the total phenols content, while in the second, the shoot Mn and Zn contents increased.

Nitrogen application between the first and the second harvest may be needless. In general, the phytochemical accumulation and antioxidant activity of the leaves decreased in the second harvest.

The findings indicate that combined application of ammonium nitrate and organic compost is a strategy to reduce inorganic nitrogen application.

## Figures and Tables

**Table 1:** Effects of the addition of organic compost and inorganic nitrogen fertigation on shoot dry weight and fresh yield of coriander on two successive harvests.

Treatment	Shoot Dry Weight	Fresh Yield	Total Fresh Yield
	(g/plant)	(kg m^−2^)	
1st harvest			
S ^1^	-	0.09 f	-
OC	0.47 ^z^c	1.55 c	
OC + AS	0.62 b	1.95 b	
OC + AN	0.72 a	2.43 a	
2nd harvest			
S ^1^	-	-	-
OC	0.43 d	1.21 d	2.76 b
OC + AS	0.34 e	0.97 d	2.92 ab
OC + AN	0.39 d	1.20 d	3.63 a
Significance			
OC + Nitrogen	**	**	**
Harvest	*	***	-
Interaction	***	**	-

^z^—means followed by different letter within a column are significantly different at *p* ≤ 0.05 (Duncan test). Means followed by different letters within a column are significantly different. *, **, and *** significant at *p* < 0.05, 0.01, and 0.001 levels, respectively. S—unfertilized soil, OC—organic compost, OC + AS—organic compost + 60 kg of N ha^−1^ applied as ammonium sulfate, OC + AN—organic compost + 60 kg N ha^−1^ applied as ammonium nitrate. ^1^ In the unfertilized soil, no shoot samples were collected because the plants withered and died.

**Table 2:** Effect of organic compost combined with inorganic nitrogen fertigation on shoot nutrients’ concentration.

Treatment	Shoot Macronutrients (mg·g^−1^)					Shoot Micronutrients (μg·g^−1^)				
	N	P	K	Ca	Mg	Fe	B	Cu	Mn	Zn
1st harvest										
OC	4.62 d ^z^	0.62 c	5.17 a	0.73 b	0.43 c	115.5 a	33.5 b	15.5 b	131.0 c	84.5 e
OC + AS	5.44 bc	0.67 b	5.21 a	0.73 b	0.41 c	129.5 a	31.0 b	15.5 b	142.5 c	105.5 d
OC + AN	5.41 c	0.63 bc	5.48 a	0.75 b	0.40 c	133.5 a	32.5 b	8.4 c	84.0 d	81.5 e
2nd harvest										
OC	5.62 b	0.73 a	5.44 a	0.85 a	0.48 ab	133.0 a	35.0 a	15.5 b	130.5 c	182.5 c
OC + AS	6.76 a	0.67 b	5.37 a	0.89 a	0.49 ab	137.5 a	39.5 a	17.0 b	302.0 a	200.5 a
OC + AN	6.68 a	0.68 b	5.49 a	1.00 a	0.55 a	131.5 a	39.0 a	22.0 a	243.0 b	190.5 b
Significance										
OC + Nitrogen	**	NS	NS	NS	*	NS	NS	NS	***	***
Harvest	***	**	NS	*	*	NS	*	***	***	***
Interaction	NS	*	NS	NS	NS	NS	NS	***	***	***

^z^—means followed by different letters within a column are significantly different. *, **, and *** significant at *p* < 0.05, 0.01, and 0.001 levels, respectively. NS—not significant. OC—organic compost, OC + AS—organic compost + 60 kg of N ha^−1^ applied as ammonium sulfate, OC + AN—organic compost + 60 kg N ha^−1^ applied as ammonium nitrate.

**Table 3:** Effect of organic compost combined with inorganic nitrogen fertigation on shoot N, P, K, Ca, and Mg uptake.

Treatment	Shoot Macronutrients (mg/plant)				
	N	P	K	Ca	Mg
1st harvest					
OC	21.7 d ^z^	2.91 b	24.3 c	3.43 c	2.02 b
OC + AS	33.7 b	4.15 a	32.3 b	4.52 b	2.50 a
OC + AN	38.9 a	4.53 ab	39.6 a	5.40 a	2.88 a
2nd harvest					
OC	24.9 d	3.13 b	21.2 c	3.65 c	2.01 b
OC + AS	21.1 d	2.22 c	18.3 e	3.00 d	1.70 c
OC + AN	26.1 c	2.27 c	23.1 c	3.90 c	2.10 b
Significance					
OC + Nitrogen	**	NS	*	*	*
Harvest	*	***	***	*	*
Interaction	NS	***	*	NS	NS

^z^—means followed by different letters within a column are significantly different. *, **, and *** significant at *p* < 0.05, 0.01, and 0.001 levels, respectively. NS—not significant. OC—organic compost, OC + AS—organic compost + 60 kg of N ha^−1^ applied as ammonium sulfate, OC + AN—organic compost+ 60 kg N ha^−1^ applied as ammonium nitrate. Means followed by different letter within a column are significantly different at *p* ≤ 0.05.

**Table 4:** Effect of organic compost combined with inorganic nitrogen fertigation on leaf photosynthetic pigments content.

Treatment	Photosynthetic Pigments (mg/100 g FW)			
	Total Chl	Chl a	Chl b	Cc
1st harvest				
OC	96.42 d ^z^	61.40 d	35.02 c	46.16 b
OC + AS	138.91 bc	80.72 c	58.19 a	83.42 a
OC + AN	140.52 bc	85.61 c	54.90 ab	72.88 a
2nd harvest				
OC	121.48 c	74.00 cd	47.48 b	47.80 b
OC + AS	162.01 b	103.02 b	58.99 a	41.79 b
OC + AN	187.14 a	123.94 a	63.20 a	50.30 b
Significance				
OC + Nitrogen	***	***	***	***
Harvest	***	***	*	***
Interaction	NS	NS	NS	***

^z^—means followed by different letters within a column are significantly different. *, and *** significant at *p* < 0.05, 0.01, and 0.001 levels, respectively. NS—not significant. OC—organic compost, OC + AS—organic compost + 60 kg of N ha^−1^ applied as ammonium sulfate, OC + AN—organic compost + 60 kg N ha^−1^ applied as ammonium nitrate. Chl a—chlorophyl a, Chl b—chlorophyl b, Cc—Carotenoids.

**Table 5:** Effect of organic compost combined with inorganic nitrogen fertigation on shoot TPC, AsA, Proline, PDH, DPPH, and FRAP.

Treatment	TPC	AsA	Proline	PDH	DPPH	FRAP
	mgGAE/100 g FW	mg/100 g FW	mg/100 g FW	nmol min^−1^/mg	mgGAE/100 g FW	mgTrolox/g FW
1st harvest						
OC	174.7 b ^z^	15.1 b	45.8 a	4.7 c	45.61 a	178.9 a
OC + AS	280.4 a	12.7 bc	49.7 a	5.9 bc	7.84 b	196.8 a
OC + AN	136.8 c	11.5 c	17.4 b	2.7 d	45.34 a	173.5 a
2nd harvest						
OC	60.1 e	22.4 a	15.1 c	2.0 d	9.6 b	83.7 c
OC + AS	68.0 e	23.0 a	16.7 c	9.9 a	11.1 b	100.6 bc
OC + AN	90.3 d	22.9 a	14.5 c	6.7 b	10.8 b	127.8 b
Significance						
OC + Nitrogen	***	NS	***	***	***	*
Harvest	***	**	***	***	***	***
Interaction	***	**	***	***	***	***

^z^—means followed by different letters within a column are significantly different. *, **, and *** significant at *p* < 0.05, 0.01, and 0.001 levels, respectively. NS—not significant. OC—organic compost, OC + AS—organic compost + 60 kg of N ha^−1^ applied as ammonium sulfate, OC + AN—organic compost + 60 kg N ha^−1^ applied as ammonium nitrate.

## Data Availability

Not applicable.

## References

[1] Britannica, The Editors of Encyclopaedia “Coriander”. Encyclopedia Britannica. https://www.britannica.com/plant/coriander.

[2] Almeida D. (2006). Manual for Horticultural Crops.

[3] Freitas A.D., Bernardes J.P., Mateus M.P., Braz N. (2015). Dimensions of Mediterranean Diet: World Cultural Heritage.

[4] Santos J., Herrero M., Mendiola J.A., Oliva-Teles M.T., Ibáñez E., Delerue-Matos C., Oliveira M.B.P.P. (2014). Fresh-cut aromatic herbs: Nutritional quality stability during shelf-life. LWT-Food Sci. Technol..

[5] Nguyen D.T.P., Kitayama M., Lu N., Takagaki M. (2019). Improving secondary metabolite accumulation, mineral content, and growth of coriander (*Coriandrum sativum* L.) by regulating light quality in a plant factory. J. Hortic. Sci. Biotechnol..

[6] Carrubba A. (2009). Nitrogen fertilisation in coriander (*Coriandrum sativum* L.): A review and meta-analysis. J. Sci. Food Agric..

[7] Abdollahi F., Salehi A., Shahabi R., Rahimi A. (2016). Effect of different nitrogen sources on vegetative traits, grain yield and essential oil yield of coriander (*Coriandrum sativum*). Cercet. Agron. Mold..

[8] Scheible W.-R., Morcuende R., Czechowski T., Fritz C., Osuna D., Palacios-Rojas N., Schindelasch D., Thimm O., Udvardi M.K., Stitt M. (2004). Genome-Wide reprogramming of primary and secondary metabolism, protein synthesis, cellular growth processes, and the regulatory infrastructure of Arabidopsis in response to nitrogen. Plant Physiol..

[9] Argyropoulou K., Salahas G., Hela D., Papasavvas A. (2015). Impact of nitrogen deficiency on biomass production, morphological, and biochemical characteristics of sweet basil (*Ocimum basilicum* L.) Plants, cultivated aeroponically. J. Int. Sci. Publ..

[10] Xu C., Mou B. (2016). Responses of spinach to salinity and nutrient deficiency in growth, physiology, and nutritional value. J. Am. Soc. Hortic. Sci..

[11] Zhou W., Liang X., Zhang Y., Li K., Jin B., Lu L., Jin C., Lin X. (2019). Reduced nitrogen supply enhances the cellular antioxidant potential of phenolic extracts through alteration of the phenolic composition in lettuce (*Lactuca sativa* L.). J. Sci. Food Agric..

[12] Leser C., Treutter D. (2005). Effects of nitrogen supply on growth, contents of phenolic compounds and pathogen (scab) resistance of apple trees. Physiol. Plantarum..

[13] Akula R., Ravisnankar G.A. (2011). Influence of abiotic stress signals on secondary metabolites in plants. Plant Signal. Behav..

[14] Gutiérrez-Gamboa G., Portu J., López R., Santamaría P., Garde-Cerdán T. (2018). Elicitor and nitrogen applications to Garnacha, Graciano and Tempranillo vines: Effect on grape amino acid composition. J. Sci. Food Agric..

[15] Machado R.M.A., Alves-Pereira I., Lourenço D., Ferreira R.M.A. (2020). Effect of organic compost and inorganic nitrogen fertigation on spinach growth, phytochemical accumulation, and antioxidant activity. Heliyon.

[16] Li J., Zhu Z., Gerendás J. (2008). Effects of nitrogen and sulfur on total phenolics and antioxidant activity in two genotypes of leaf mustard. J. Plant Nutr..

[17] Machado R.M.A., Alves-Pereira I., Robalo M., Ferreira R. (2021). Effect of municipal solid waste compost rate supplemented with inorganic nitrogen on physicochemical soil characteristics, plant growth, nitrate content and antioxidant activity in spinach. Horticulturae.

[18] Graham R.F., Sam E.W., Pittelkow C.M. (2017). Comparison of organic and integrated nutrient management strategies for reducing soil N_2_O emissions. Sustainability.

[19] Donega M.A., Mello S.C., Moraes R.M., Cantrell C.L. (2013). Nutrient uptake, biomass yield and quantitative analysis of aliphatic aldehydes in cilantro plants. Ind. Crop. Prod..

[20] Trani P.E., Purquerio L.F.V., Figueiredo G.B., Tivelli S.W., Blat S.F. (2014). Calagem e Adubação da Alface, Almeirão, Agrião-d’água, chicória, coentro, espinafre e Rúcula.

[21] Mills H.A., Jones J.R. (1996). Plant Analysis Handbook II. A Practical Sampling, Preparation Analysis and Interpretation Guide.

[22] Penn C.J., Camberato J.J. (2019). A critical review on soil chemical processes that control how soil pH affects phosphorus availability to plants. Agriculture.

[23] Neina D. (2019). The role of soil pH in plant nutrition and soil remediation. Appl. Environ. Soil Sci..

[24] Mortvedt J.J., Cox F.R., Shuman L.M., Welch R.M. (1991). Micronutrients in Agriculture.

[25] Kumar V., Yadav D.V., Yadav D.S. (1990). Effects of nitrogen sources and copper levels on yield, nitrogen, and copper contents of wheat (*Triticum aestivum* L.). Plant Soil.

[26] Gerendás J., Zhu Z.J., Bendixen R., Ratcliffe R.G., Sattelmacher B. (1997). Physiological and biochemical processes related to ammonium toxicity in higher plants. Z. Pflanz. Bodenkd..

[27] Roosta H.R., Schjoerring J.K. (2007). Effects of ammonium toxicity on nitrogen metabolism and elemental profile of cucumber plants. J. Plant Nutr..

[28] Nguyen D.T., Lu N., Kagawa N., Kitayama M., Takagaki M. (2020). Short-term root-zone temperature treatment enhanced the accumulation of secondary metabolites of hydroponic coriander (*Coriandrum sativum* L.) grown in a plant factory. Agronomy.

[29] Bajpai M., Mishra A., Prakash D. (2005). Antioxidant and free radical scavenging activities of some leafy vegetables. Int. J. Food Sci. Nutr..

[30] Kopsell D.A., Kopsell D.E., Curran-Celentano J. (2007). Carotenoid pigments in kale are influenced by nitrogen concentration and form. J. Sci. Food Agric..

[31] Di Mola I., Ottaiano L., Cozzolino E., Senatore M., Sacco A., El-Nakhel C., Rouphael Y., Mori M. (2020). *Trichoderma* spp. and mulching films differentially boost qualitative and quantitative aspects of greenhouse lettuce under diverse N conditions. Horticulturae.

[32] Becker C., Urlić B., Jukić Špika M., Kläring H.P., Krumbein A., Baldermann S., Goreta S., Perica S., Schwarz D. (2015). Nitrogen limited red and green leaf lettuce accumulate flavonoid glycosides, caffeic acid derivatives, and sucrose while losing chlorophylls, β-carotene and xanthophylls. PLoS ONE.

[33] Chenard C.H., Kopsell D.A., Kopsell D.E. (2005). Nitrogen concentration affects nutrient and carotenoid accumulation in parsley. J. Plant Nutr..

[34] Divya P., Puthusseri B., Neelwarne B. (2012). Carotenoid content, its stability during drying and the antioxidant activity of commercial coriander (*Coriandrum sativum* L.) varieties. Food Res. Int..

[35] Nambiar V.S., Sharma M. (2014). Carotene content of coriander leaves (*Coriandrum sativum*), amaranth, red (*Amaranthus* Sp.), green garlic (Allium sativum) and mogri (Raphanus caudatus) and its products. J. Appl. Pharm. Sci..

[36] Ngamwonglumlert L., Devahastin S., Chiewchan N., Raghavan V. (2020). Plant carotenoids evolution during cultivation, postharvest storage, and food processing: A review. Compr. Rev. Food Sci. Food Saf..

[37] Nguyen P.M., Niemeyer E.D. (2008). Effects of nitrogen fertilization on the phenolic composition and antioxidant properties of basil (*Ocimum basilicum* L.). J. Agric. Food Chem..

[38] Zhu W., Lin X., Jin C., Zhang Y., Fang P. (2009). Effects of nitrogen application rates on antioxidant contents and antioxidative activities in Chinese cabbage (*Brassica chinensis* L.). J. Zhejiang Univ. (Agric. Life Sci.).

[39] Coria-Cayupaìn Y.S., Saìnchez de Pinto M.I., Nazareno M.A. (2009). Variations in bioactive substance contents and crop yields of lettuce (*Lactuca sativa* L.) cultivated in soils with different fertilization treatments. J. Agric. Food Chem..

[40] Petropoulos S., Fernandes Â., Karkanisc A., Antoniadisd V., Barros L., Ferreira I.C.F.R. (2018). Nutrient solution composition and growing season affect yield and chemical composition of *Cichorium spinosum* plants. Sci. Hortic..

[41] Munene R., Changamu E., Korir N., Joseph G.O. (2017). Effects of different nitrogen forms on growth, phenolics, flavonoids and antioxidant activity in amaranth species. Trop. Plant Res..

[42] Prinsi B., Morgutti S., Negrini N., Faoro F., Espen L. (2020). Insight into composition of bioactive phenolic compounds in leaves and flowers of green and purple basil. Plants.

[43] Hui Y., Wang J., Jiang T., Ma T., Wang R. (2021). Effect of nitrogen regulation on berry quality and flavonoids during veraison stage. Food Sci. Nutr..

[44] Amarowicz R., Cwalina-Ambroziak B., Janiak M.A., Bogucka B. (2020). Effect of N Fertilization on the content of phenolic compounds in Jerusalem artichoke (*Helianthus tuberosus* L.) tubers and their antioxidant capacity. Agronomy.

[45] Ma D., Sun D., Li Y., Wang C., Xie Y., Guo T. (2015). Effect of nitrogen fertilization and irrigation on phenolic content, phenolic acid composition, and antioxidant activity of winter wheat grain. J. Sci. Food Agric..

[46] Zhao C., Wang Z., Cui R., Su L., Sun X., Borras-Hidalgo O., Li H., Wei J., Yue Q., Zhao L. (2021). Effects of nitrogen application on phytochemical component levels and anticancer and antioxidant activities of *Allium fistulosum*. PeerJ.

[47] Bartoli C.G., Pastori G.M., Foyer C.H. (2000). Ascorbate biosynthesis in mitochondria is linked to the electron transport chain between complexes III and IV. Plant Physiol..

[48] Bergquist S.Å., Gertsson U.E., Olsson M.E. (2006). Influence of growth stage and postharvest storage on ascorbic acid and carotenoid content and visual quality of baby spinach (*Spinacia oleracea* L.). J. Sci. Food Agric..

[49] Ahmadi M., Souri M.K. (2018). Growth and mineral content of coriander (*Coriandrum sativum* L.) plants under mild salinity with different salts. Acta Physiol. Plant..

[50] Mohammadipour N., Souri M.K. (2019). Beneficial effects of glycine on growth and leaf nutrient concentrations of coriander (*Coriandrum sativum*) plants. J. Plant Nutr..

[51] Sánchez E., López-Lefebre L.R., García P.C., Rivero R.M., Ruiz J.M., Romero L. (2001). Proline metabolism in response to highest nitrogen dosages in green bean plants (*Phaseolus vulgaris* L. cv. Strike). J. Plant Physiol..

[52] Tarighaleslami M., Zarghami R., Boojar M.M.A., Oveysi M. (2012). Effects of drought stress and different nitrogen levels on morphological traits of proline in leaf and protein of corn seed (*Zea mays* L.). Am. Eurasian J. Agric. Environ. Sci..

[53] Iqbal N., Umar S., Khan N.A. (2015). Nitrogen availability regulates proline and ethylene production and alleviates salinity stress in mustard (*Brassica juncea*). J. Plant Physiol..

[54] Kiyosue T., Yoshiba Y., Yamaguchi-Shinozaki K., Shinozaki K. (1996). A nuclear gene encoding mitochondrial proline dehydrogenase, an enzyme involved in proline metabolism, is upregulated by proline but downregulated by dehydration in Arabidopsis. Plant Cell.

[55] Peng Z., Lu Q., Verma D.P. (1996). Reciprocal regulation of delta 1-pyrroline-5-carboxylate synthetase and proline dehydrogenase genes controls proline levels during and after osmotic stress in plants. Mol. Gen. Genet..

[56] Verbruggen N., Hermans C. (2008). Proline accumulation in plants: A review. Amino Acids.

[57] Szabados L., Savouré A. (2010). Proline: A multifunctional amino acid. Trends Plant Sci..

[58] Melo E.A., Filho J.M., Guerra N.B., Maciel G.R. (2003). Atividade antioxidante de extratos de coentro (*Coriandrum sativum* L.). Cienc. Tecnol. Aliment. Camp..

[59] Ghoora M.D., Haldipur A.C., Srividya N. (2020). Comparative evaluation of phytochemical content, antioxidant capacities and overall antioxidant potential of select culinary microgreens. J. Agric. Food Res..

[60] Alan O., Avci A.B., Giachino R.R. (2017). Harvest number and growing season effects on quality and health related compounds in parsley. Indian J. Pharm. Educ. Res..

[61] Corrado G., Chiaiese P., Lucini L., Miras-Moreno B., Colla G., Rouphael Y. (2020). Successive harvests affect yield, quality, and metabolic profile of sweet basil (*Ocimum basilicum* L.). Agronomy.

[62] Mandim F., Petropoulos S.A., Pinela J., Dias M.I., Giannoulis K.D., Kostić M., Soković M., Queijo B., Santos-Buelga C., Ferreira I.C.F.R. (2022). Chemical composition and biological activity of cardoon (*Cynara cardunculus* L. var. altilis) seeds harvested at different maturity stages. Food Chem..

[63] Pérez-López U., Sgherrib C., Miranda-Apodaca J., Micaelli F., Lacuestac M., Mena-Petitea A., Quartacci M.F., Muñoz-Rueda A. (2018). Concentration of phenolic compounds is increased in lettuce grown under high light intensity and elevated CO_2_. Plant Physiol. Bioch..

[64] Li Y., Kong D., Fu Y., Sussman M.R., Wu H. (2020). The effect of developmental and environmental factors on secondary metabolites in medicinal plants. Plant Physiol. Biochem..

[65] Yang L., Wen K.-S., Ruan X., Zhao Y.-X., Wei F., Wang Q. (2018). Response of Plant Secondary Metabolites to Environmental Factors. Molecules.

[66] ICT (2018). Atmospheric Sciences Water and Climate. http://www.ict.uevora.pt/g1/index.php/meteo-data/.

[67] Lichtenthaler H.K., Buschmann C. (2001). Chlorophylls and carotenoids: Measurement and characterization by UV-VIS spectroscopy. Curr. Protoc. Food Anal. Chem..

[68] Bouayed J., Hoffmann L., Bohn T. (2011). Total phenolics, flavonoids, anthocyanins and antioxidant activity following simulated gastro-intestinal digestion and dialysis of apple varieties. Bioaccessibility and potential uptake. Food Chem..

[69] Cai W.M., Tang Z.C., Tang Z.C. (1999). Plant tolerance physiology. Experimental Guide for Modern Plant Physiology.

[70] Bates L.S. (1973). Rapid determination of free proline for water stress studies. Plant Soil.

[71] Brand-Williams W., Cuvelier M.E., Berse C. (1995). Use of a free radical method to evaluate antioxidant activity. LWT-Food Sci. Technol..

[72] Costilow R.N., Cooper D. (1978). Identity of proline dehydrogenase and delta1-pyrroline-5carboxylic acid reductase in *Clostridium sporogenes*. J. Bacteriol..

[73] Lake B., Snell K., Mullock B. (1987). Preparation and characterization of microsomal fractions for studies of xenobiotic metabolism. Biochemical Toxi-cology: A Practical Approach.

[74] Lowry O.H., Roseburg N.J., Farr A.L., Randell R.J. (1951). Protein measurement with the folin phenol reagent. J. Biol. Chem..

